# LESSONS LEARNED FROM NURSING HOME CIVIL MONETARY PENALTY PROJECTS

**DOI:** 10.1093/geroni/igz038.2832

**Published:** 2019-11-08

**Authors:** Diana L Sturdevant, Kathleen C Buckwalter

**Affiliations:** University of Oklahoma, Fran and Earl Ziegler College of Nursing, Oklahoma City, Oklahoma, United States

## Abstract

Nursing homes must comply with numerous federal/state regulations to receive Medicare and Medicaid funding. Failure to comply with these regulations can result in deficiency citations, and depending on the severity of the deficiency, a resulting Civil Monetary Penalty (CMP). Through the Centers for Medicare and Medicaid Services (CMS) Civil Monetary Penalty Reinvestment Program, CMP funds are reinvested to support activities that benefit nursing home residents and that protect or improve their quality of life or quality of care. This symposium presents some of the unique challenges, successes, failures, and surprise findings from CMP-funded nursing home quality improvement projects in two, predominantly rural Midwestern states: Oklahoma and Kansas. Dr. Williams presents findings of a pilot-study testing an adaptation of a successful family caregiver telehealth support intervention in the nursing home setting and implications for future research. Dr. Sturdevant shares successes, challenges, and unanticipated results from the “It’s Not OK to Fall” project, a comprehensive, 3 year fall prevention project implemented in Oklahoma nursing homes. Lastly, Ms. Round’s paper describes the implementation and findings of a Long-term Care Leadership Academy aimed at improving leadership and team building skills of three levels of nursing home staff, including Administrators/Directors’ of Nursing, RN/LPN charge nurses and certified nursing assistants. Discussant, Dr. Kathleen Buckwalter Ph.D., FAAN, RN, will discuss how principles of nursing home culture change provides a common framework for these projects and conclude by offering suggestions on how promotion of these principles might improve the quality of care provided by nursing homes.

